# Maximizing rectal dose sparing with hydrogel: A retrospective planning study

**DOI:** 10.1002/acm2.12566

**Published:** 2019-03-19

**Authors:** Owen Paetkau, Isabelle M. Gagne, Howard H. Pai, Jacqueline Lam, Jennifer Goulart, Abraham Alexander

**Affiliations:** ^1^ Department of Physics and Astronomy University of Victoria Victoria BC Canada; ^2^ Department of Medical Physics BC Cancer – Victoria Victoria BC Canada; ^3^ Department of Radiation Oncology BC Cancer – Victoria Victoria BC Canada; ^4^ Department of Surgery University of British Columbia Vancouver BC Canada

**Keywords:** EBRT, hydrogel, prostate, rectal sparing, SpaceOAR

## Abstract

External beam radiation therapy for prostate cancer can result in urinary, sexual, and rectal side effects, often impairing quality of life. A polyethylene glycol‐based product, SpaceOAR© hydrogel (SOH), implanted into the connective tissue between the prostate gland and rectum can significantly reduce the dose received by the rectum and hence risk of rectal toxicity. The optimal way to manage the hydrogel and rectal structures for plan optimization is therefore of interest. In 13 patients, computerized tomography (CT) scans were taken pre‐ and post‐SpaceOAR© implant. A prescription of 60 Gy in 20 fractions was planned on both scans. Six treatment plans were produced per anonymized dataset using either a structure of rectum plus the hydrogel, termed composite rectum wall (CRW), or rectal wall (RW) as an inverse optimization structure and intensity modulated radiotherapy (IMRT) or volumetric modulated arc therapy (VMAT) as a treatment technique. Dose‐volume histogram metrics were compared between plans to determine which optimization structure and treatment technique offered the maximum rectal dose sparing. RW structures offered a statistically significant decrease in rectal dose over CRW structures, whereas the treatment technique (IMRT vs VMAT) did not significantly affect the rectal dose. There was improvement seen in bladder and penile bulb dose when VMAT was used as a treatment technique. Overall, treatment plans using the RW optimization structure offered the lowest rectal dose while VMAT treatment technique offered the lowest bladder and penile bulb dose.

## 1 INTRODUCTION

Prostate cancer is the most common noncutaneous malignancy in Canadian men, representing 21% of new cancer cases and 10% of cancer deaths in men in 2017.^1^ Standard treatment options for localized disease include surgery, external beam radiotherapy (EBRT), and brachytherapy, with many men opting for EBRT. Potential toxicities of EBRT can include rectal, urinary, and sexual dysfunction due to the proximity of the rectum, bladder, and penile bulb/neurovascular bundles to the prostate.^2^ The rectum is the dose‐limiting organ in prostate cancer external beam irradiation due to its proximity to the prostate, with the anterior rectal wall often falling within the planning target volume.^3^, ^4^, ^5^, ^6^, ^7^ In recent years a number of products have been developed to spare the rectum during radiotherapy. One such innovation is SpaceOAR© hydrogel (SOH), a polyethylene glycol‐based product, that is injected between the rectum and the prostate to displace the prostate away from the rectum. The physical shift of the rectum allows a greater proportion of the organ to be spared high dose and, in a randomized trial, has resulted in reduced rectal toxicity and improved quality of life (QOL).^8^, ^9^, ^10^, ^11^, ^12^, ^13^

The SOH has been shown to reduce the rectal dose in patients receiving both volumetric modulated arc therapy (VMAT)^11^, ^14^, ^15^ and intensity modulated radiotherapy (IMRT).^8^, ^10^, ^11^, ^12^, ^16^ Studies have compared VMAT and IMRT treatment techniques in external beam prostate cancer treatment, indicating similar results for prostate coverage. In many studies, the dose to organs at risk (OAR), including the rectum, bladder, and penile bulb, was decreased when using VMAT over IMRT.^17^, ^18^ However, one planning study shows an exception in which rectal dose was lower with application of IMRT^19^ compared to VMAT. The insertion of SOH between the rectum and the prostate may alter dose between treatment techniques. Current SOH studies are split between treatment techniques.

The generation of IMRT or VMAT plans involves an inverse planning optimization, through a series of dosimetric constraints on anatomical structures and regions within a set of radiotherapy planning computed tomography (CT) scans. In previous studies, different definitions of the rectal avoidance structure have been used during optimization to minimize the true rectal organ dose. To date, the rectum avoidance structure, which can be defined as either a solid form or a wall (i.e., rectal wall thickness of 3 mm, excluding the lumen) organ delineated from the anus or bottom of the ischial tuberosities to the rectosigmoid junction, has been commonly employed during optimization in SOH studies.^10^, ^11^, ^14^, ^15^ More recently, a fabricated structure, the composite rectal (CR), has been proposed.^16^ This structure can be generated by combining the rectum with the hydrogel before extracting a wall structure (i.e., rectum + hydrogel, thickness of 3 mm excluding the lumen). The hydrogel is difficult to contour due to low contrast between rectum and SOH on CT scans, therefore the CR structure may offer a simpler alternative. Additionally, it has been suggested by te Velde et al.^16^ that the CR may serve as an alternative rectal organ optimization structure. Optimization with each of these structures offers a varying degree of rectal dose reduction.

The aims of the present study were to firstly evaluate the ideal optimization structure rectal wall vs composite rectum wall (RW vs CRW) in the setting of SOH for hypofractionated EBRT and secondly, to test whether the VMAT technique offers additional rectal sparing compared to IMRT. In this regard, IMRT and VMAT treatment plans for 60 Gy in 20 fractions were generated using anonymized CT datasets from patients with implanted SOH, using RW and CRW in the optimization, and organ at risk (OAR) doses were compared. The treatment plans were examined to determine which combination of optimization structure (RW or CRW) and treatment technique (VMAT or IMRT) resulted in the lowest rectal dose distribution.

## 2 MATERIALS AND METHODS

### 2.A Hydrogel implant

The anonymized CT datasets of thirteen prostate cancer patients who were implanted with 10 cc of SpaceOAR hydrogel between the prostate and the rectum were selected for this institutional research ethics board approved retrospective planning study. All patients receiving the SOH also had three to four gold fiducial markers implanted via a trans‐perineal technique prior to gel placement. The CT datasets consisted of a pre‐SOH and post‐SOH planning CT scans for each patient. The pre‐SOH planning CT scan was obtained with a comfortably full bladder and empty rectum 30 to 60 min prior to implantation of fiducial markers and SOH. Patients were given specific instructions to drink 750 ml of water within 15 min, 1 h prior to their pre‐SOH CT scan and to perform a micro‐enema 2 to 3 h prior to their appointment. One week later patients underwent a post‐SOH planning CT scan as well as a pelvic MRI with the same bladder and bowel preparation instructions. The MR images were registered to the post‐SOH planning CT images, using fusion to the gold fiducial markers, and used to assist with contouring the SOH, rectum, and prostate gland.

### 2.B Structure of interest contours

**Figure 1:**
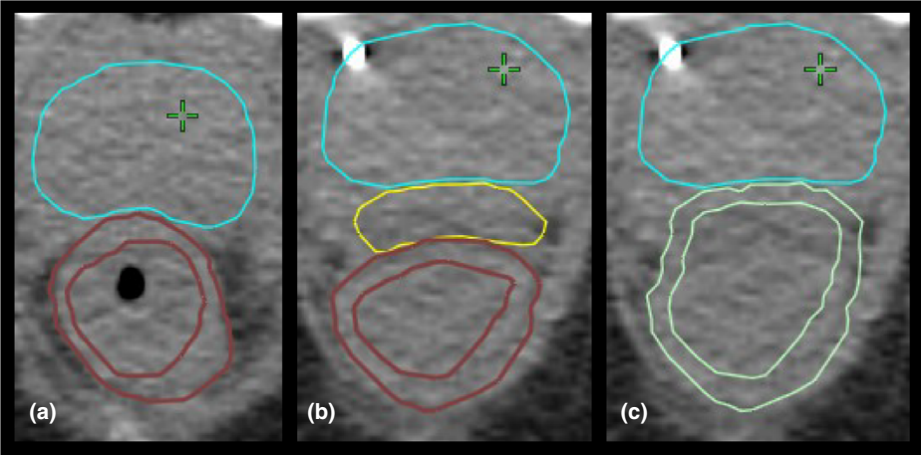
Computed tomography scan with clinical target volume (CTV) and rectum structures: (a) pre‐SpaceOAR© hydrogel (SOH) with CTV and rectal wall (RW), (b) post‐SOH with CTV, SOH and RW, and (c) post‐SOH with CTV and composite rectum wall.

A set of target and OARs for optimization and plan evaluation purposes were defined and peer‐reviewed by a group of genitourinary radiation oncologists. Clinical target volume (CTV) was defined as the prostate gland and proximal 1 cm of seminal vesicles. The planning target volume (PTV) was defined as the CTV with margins of 7 mm in all directions except for a 5 mm margin in the posterior direction. Rectum was contoured as a solid organ from the rectosigmoid junction to the ischial tuberosities, and the cranial‐caudal length was kept consistent from pre‐ to post‐SOH. Composite rectum (CR) was defined as hydrogel plus rectum with manual editing to smooth jagged contours. RW and CRW structures were extracted using an inner wall margin of 3 mm (Fig. 1). Bladder was contoured with a bladder wall (BW) extracted using an inner wall margin of 3 mm. Femoral heads were contoured separately from the top of femoral head to the lesser trochanter. The penile bulb was contoured as the bulbous spongiosum below the GU diaphragm and proximal to the penile shaft.

### 2.C Treatment plans

A total of six hypofractionated, 60 Gy in 20 fractions, treatment plans were created for each patient using Eclipse version 13.6 with the final dose calculation performed with the anisotropic analytical algorithm (AAA) version 11.0.31. This included a pre‐SOH plan, and two post‐SOH plans using either the RW or the CRW as the optimization structure. These plans were created using both IMRT (dose volume optimizer version 11.0.31) and single arc VMAT (progressive resolution optimizer version 11.0.31) treatment techniques to produce the treatment plans listed in Table 1. Most IMRT plans were created using five angle beam arrangements (0°, 50°, 100°, 260°, and 310°). Two additional beam angles (155° and 205°) were added to plans when hot spots in the subcutaneous tissues exceeded planning guidelines with a five beam arrangement.

**Table 1:** The six treatment plans created to compare optimization structure and treatment technique

Treatment plan	Acronym	Hydrogel	Treatment technique	Structure
1	Pre_IMRT	No	IMRT	Rectum
2	CRW_IMRT	Yes	IMRT	Composite rectum
3	RW_IMRT	Yes	IMRT	Rectum
4	Pre_VMAT	No	VMAT	Rectum
5	CRW_VMAT	Yes	VMAT	Composite rectum
6	RW_VMAT	Yes	VMAT	Rectum

CRW: composite rectum wall; IMRT: intensity modulated radiotherapy; RW: rectal wall; VMAT: volumetric modulated arc therapy.

A plan optimization was deemed successful when the objectives listed in Table 2 were met using a plan normalization adjustment of less than ±0.5% following final dose calculation with AAA. Treatment plans which meet all OAR clinical objectives were difficult to produce with no plan normalization adjustment. In a clinical setting, plan normalization may vary up to 5% to meet required goals. Restricting the plan normalization to ±0.5% limited its impact on the treatment plan comparison and ensured the correct balance between target coverage and OAR was achieved mainly during the optimization stage. The small adjustment to plan normalization limited the effect of plan normalization on treatment plans, creating a more difficult task for the planner to achieve the clinical goals set in Table 2. The clinical objectives, which are routinely utilized at BC Cancer – Victoria for prostate 60 Gy hypofractionated radiotherapy, were adapted from the PROFIT^20^ and the CHHiP^21^ study dosimetric objectives. The structures RW17.5 and BW17.5 in Table 2 were used solely to evaluate the quality of the plans and were contoured from 17.5 mm superiorly to the cranial border of the PTV to 17.5 mm inferiorly to the caudal border of the PTV. All optimizations were performed by a single planner. CRW or RW dose‐volume constraints were adjusted during the optimization on a patient by patient basis to produce the lowest rectal dose possible. For the purpose of this study, the rectal volumes receiving 60, 55, 50, 46, and 37 Gy were compared. Pre‐SOH and post‐SOH CTV, PTV and OAR volumes were also compared along with CTV and PTV mean doses and bladder volumes receiving 60, 55, 50, 46, and 37 Gy.

**Table 2:** Planning goals for both intensity modulated radiotherapy (IMRT) and volumetric modulated arc therapy (VMAT) plans

Structure	Metric (cGy)	Volume
PTV	V5700	≥99%
	V6300	1.00 cc
CTV	V6000	≥99%
RW17.5	V4600	≤30%
	V3700	≤50%
BW17.5	V4600	≤30%
	V3700	≤50%
Left femoral head	V4300	≤2.5%
Rightt femoral head	V4300	≤2.5%
Penile bulb	V4166	≤50%

CTV: clinical target volume; PTV: planning target volume.

### 2.D Statistical analysis

The statistical testing was done using the nonparametric Wilcoxon signed rank test to compare different plan types as well as observe the change in volume between the pre‐ and post‐SOH CT scans. The tests were two‐sided and considered significant at *P *< 0.01.

## 3 RESULTS

Volume statistics for structures in the pre‐ and post‐SOH CT datasets are summarized in Table 3. The majority of structures showed no significant difference between the CT scans with the exception of the PTV which showed a significant difference between pre‐ and post‐SOH volumes (*P* = 0.006). The composite rectum volume was also statistically different from the summed individual rectum and SOH volumes (*P* = 0.001) due to smoothing of the edges of the composite rectum structure.

**Table 3:** Mean, standard deviation, and range of volumes of target and organ at risks contoured on both pre‐ and post‐SpaceOAR© hydrogel computerized tomography dataset

Structure	Prgel volume (cc)	Postgel volume (cc)	*P*‐value
CTV	34 ± 7 (25–46)	35 ± 8 (24–49)	0.04
PTV	96 ± 14 (78–120)	102 ± 15 (81–128)	**0.006**
SpaceOAR gel	–	11 ± 2 (8–12)	–
Bladder	327 ± 197 (154–827)	286 ± 88 (152–431)	0.77
Bladder wall	73 ± 28 (44–144)	66 ± 13 (43–91)	0.42
Penile bulb	3 ± 1 (1–4)	2 ± 1 (1–4)	0.60
Rectum	85 ± 35 (45–150)	77 ± 18 (46–103)	0.35
Rectum wall	36 ± 9 (23–56)	36 ± 5 (28–45)	0.65
Composite rectum	–	87 ± 19 (58–117)	–
Composite rectum wall	–	40 ± 5 (31–47)	–

CTV: clinical target volume; PTV: planning target volume.

Bold values indicate statistical significance below the threshhold of *P* < 0.01.

Comparisons between all six treatment plans (two pre‐ and four post‐SOH plans) are shown in Table 4 while Table 5 show the p‐values associated with the various treatment plans for the rectal dosimetry. There were no significant differences in mean CTV and PTV coverage with regards to SOH implant, optimization structure, and delivery technique. The bladder dose was unaffected by the SOH implant for all five dose levels examined in both IMRT and VMAT plans. Overall, VMAT technique yielded treatment plans with lower bladder dose compared to IMRT, with BV55 Gy, BV50 Gy, and BV46 Gy achieving statistical significance (*P* < 0.007). Similarly, the mean penile bulb (PB) dose was found to be unaffected by the SOH implant (*P* > 0.24) while there was statistically significant improvement found in VMAT plans compared to IMRT plans (*P* < 0.04).

**Table 4:** Mean, standard deviation and range of dose‐volume metrics for **clinical** target volume (CTV), planning target volume (PTV), penile bulb bladder and rectum

Plan/metric	Pre_IMRT	CRW_IMRT	RW_IMRT	Pre_VMAT	CRW_VMAT	RW_VMAT
CTV mean dose (%)	102.0 ± 0.7 (100.5–102.9)	102.4 ± 0.3 (101.9–102.7)	102.4 ± 0.3 (102.1–102.8)	102.1 ± 0.2 (101.8–102.6)	102.1 ± 0.1 (101.8–102.3)	102.0 ± 0.1 (101.7–102.1)
PTV mean dose (%)	101.6 ± 0.7 (100.1–102.4)	101.8 ± 0.2 (101.4–102.3)	101.8 ± 0.2 (101.5–102.1)	101.7 ± 0.1 (101.6–101.9)	101.8 ± 0.1 (101.5–101.9)	101.8 ± 0.2 (101.5–102.3)
PB mean dose (%)	15.2 ± 12.9 (5.8–51.9)	16.8 ± 16.9 (5.4–66.9)	16.9 ± 17.0 (5.7–67.7)	14.0 ± 11.7 (4.7–45.5)	16.4 ± 17.4 (5.0–68.4)	16.6 ± 17.3 (5.0–68.0)
BV60 Gy (%)	3.4 ± 1.3 (1.3–5.6)	3.7 ± 1.5 (0.9–6.8)	3.6 ± 1.6 (0.9–6.5)	3.1 ± 1.4 (1.2–6.4)	3.6 ± 1.6 (1.0–7.4)	3.6 ± 1.6 (1.0–7.4)
BV55 Gy (%)	6.3 ± 2.9 (2.1–13.5)	6.6 ± 2.8 (1.8–12.4)	6.6 ± 2.8 (1.7–12.2)	5.4 ± 2.4 (2.0–10.7)	5.9 ± 2.5 (1.7–11.5)	5.9 ± 2.5 (1.6–11.4)
BV50 Gy (%)	8.4 ± 3.9 (2.8–17.8)	8.8 ± 3.8 (2.5–16.9)	8.8 ± 3.7 (2.4–16.7)	7.3 ± 3.2 (2.6–13.7)	7.8 ± 3.1 (2.4–14.9)	7.7 ± 3.2 (2.2–14.7)
BV46 Gy (%)	10.1 ± 4.7 (3.3–20.9)	10.6 ± 4.6 (3.2–20.9)	10.7 ± 4.6 (3.1–21.0)	9.0 ± 3.9 (3.2–16.4)	9.5 ± 3.7 (3.1–17.9)	9.4 ± 3.8 (2.8–17.8)
BV37 Gy (%)	15.7 ± 7.6 (4.7–29.8)	15.8 ± 6.3 (6.0–29.2)	16.0 ± 6.2 (5.8–29.5)	14.5 ± 6.8 (5.0–24.8)	15.2 ± 5.6 (6.0–27.2)	14.7 ± 5.6 (5.1–26.2)
RV60 Gy (%)	1.1 ± 0.9 (0.0–2.5)	0.2 ± 0.3 (0.0–0.8)	0.2 ± 0.4 (0.0–1.4)	1.6 ± 1.7 (0.0–6.2)	0.3 ± 0.5 (0.0–1.8)	0.3 ± 0.4 (0.0–1.3)
RV55 Gy (%)	5.4 ± 4.6 (0.5–16.5)	1.6 ± 2.4 (0.0–7.8)	1.2 ± 2.0 (0.0–6.6)	5.2 ± 4.0 (0.8–13.5)	1.5 ± 2.1 (0.0–6.7)	1.1 ± 1.7 (0.0–5.7)
RV50 Gy (%)	8.5 ± 6.6 (1.3–22.8)	3.3 ± 4.2 (0.0–14.1)	2.4 ± 3.6 (0.0–11.6)	8.3 ± 5.4 (2.4–19.0)	3.1 ± 3.3 (0.1–11.0)	2.2 ± 3.1 (0.0–9.8)
RV46 Gy (%)	11.0 ± 7.9 (2.2–26.7)	5.3 ± 5.5 (0.2–18.8)	3.9 ± 4.9 (0.1–16.3)	11.2 ± 6.5 (4.3–23.6)	5.1 ± 4.3 (0.6–14.8)	3.4 ± 4.3 (0.1–13.4)
RV37 Gy(%)	18.2 ± 10.3 (5.0–35.6)	12.4 ± 8.0 (1.7‐29.1)	10.3 ± 8.1 (1.2‐27.5)	21.6 ± 8.5 (11.3‐37.4)	15.7 ± 7.3 (5.4‐26.9)	10.4 ± 7.9 (2.2‐25.2)

CRW: composite rectum wall; IMRT: intensity modulated radiotherapy; RW: rectal wall; VMAT: volumetric modulated arc therapy.

**Table 5:** The *P*‐values from plan to plan comparisons of the rectal metrics

Treatment plans		RV60 Gy	RV55 Gy	RV50 Gy	RV46 Gy	RV37 Gy
Pre_IMRT	CRW_IMRT	**0.002**	**0.003**	**0.004**	**0.004**	**0.007**
Pre_IMRT	RW_IMRT	**0.002**	**0.002**	**0.002**	**0.002**	**0.002**
CRW_IMRT	RW_IMRT	0.890	**0.002**	**0.002**	**0.002**	0.019
Pre_VMAT	CRW_VMAT	**0.006**	**0.002**	**0.002**	**0.002**	**0.002**
Pre_VMAT	RW_VMAT	**0.003**	**0.002**	**0.002**	**0.002**	**0.002**
CRW_VMAT	RW_VMAT	0.859	**0.002**	**0.002**	**0.002**	**0.002**
Pre_IMRT	Pre_VMAT	0.917	0.650	0.807	0.650	0.033
CRW_IMRT	CRW_VMAT	0.139	0.814	0.917	0.917	0.039
RW_IMRT	RW_VMAT	0.091	0.594	0.807	0.553	0.753

CRW: composite rectum wall; IMRT: intensity modulated radiotherapy; RW: rectal wall; VMAT: volumetric modulated arc therapy.

Bold values indicate statistical significance below the threshhold of *P* < 0.01.

Both IMRT and VMAT post‐SOH plans resulted in rectal dose‐volume reductions of greater than 25% for each metric compared to respective pre‐SOH plans. At the prescription dose level, average volume reductions were greater than 80% for all post‐SOH plans while they ranged from 28% to 54% at 37 Gy, the lowest dose level evaluated in this study. For both IMRT and VMAT techniques, the use of RW structure in the optimization resulted in significantly lower rectal volumes at doses of 55, 50, and 46 Gy while there was no significant reduction at the prescription dose level of 60 Gy (P_IMRT_ = 0.890, P_VMAT_ = 0.859). The use of RW in the optimization compared to CRW did not result in statistically significant reductions in rectal volumes receiving 37 Gy when IMRT (*P* = 0.019) was selected as the technique of choice but did result in significant dose reductions for VMAT (*P* = 0.002).

## 4 DISCUSSION

SOH has been incorporated into the radiotherapeutic management of prostate cancer in numerous cancer centers as a result of the proven benefits in reducing rectal toxicity and improving QOL.^9^, ^22^ Optimal treatment planning techniques are essential to maximize the benefits of SOH. Contoured structures or volumes are used in IMRT or VMAT inverse planning optimization processes to conform dose to the PTV while minimizing the dose to OARs such as the rectum and the bladder. As such, the method in which these optimization structures are contoured and utilized will affect the dosimetric profile of the treatment plan, and therefore the OAR dose. To date, many different optimization structures have been utilized in SOH studies to reduce rectal dose. Rajecki et al.^15^ used a control region to shape the dose distribution in the posterior region of the prostate, te Velde et al.^16^ chose to contour the SOH and rectal wall together as the CRW to minimize the dose to the true rectum. The SOH is difficult to contour due to low contrast between rectum and SOH on CT scans and as such a registered MR image is required to identify the SOH. This offers new challenges due to organ motion between the CT and MRI as such, the CRW structure offers a simpler contouring process. Finally, numerous studies used the rectum structure to optimize and evaluate the rectal dose.^8^, ^10^, ^14^ In the present study, IMRT and VMAT treatment plans were generated using CRW and RW contours. The results of the present study demonstrate that SOH resulted in significant reductions in rectal dose regardless of planning and contouring technique, but that the effect was most marked when optimization was performed with RW contours. Although the VMAT technique did not result in significant reductions in rectal dose compared to static field IMRT, it did offer better bladder and penile bulb sparing and conformity of intermediate dose.

Many different dose‐fractionation schedules and planning techniques have been utilized for prostate cancer radiotherapy. The pivotal SOH trial by Mariados et al.^8^ used an IMRT technique, with a dose of 79.2 Gy in 44 fractions, and rectal doses were evaluated using rectum as a whole solid organ. In the present study, treatment plans were generated using a hypofractionated prescription, 60 Gy in 20 fractions, which has now become a standard option.^20^, ^21^, ^23^ The PROFIT protocol has been adopted clinically by many Canadian centers. This protocol evaluates rectal doses using a RW structure as opposed to the whole organ. As such RW endpoints depicted in Table 2 were used in the present study as planning goals for the rectum.

**Figure 2:**
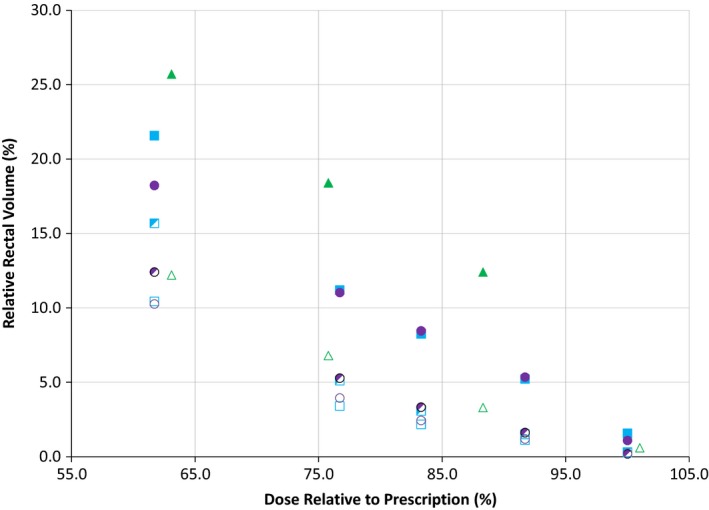
Mean rectal dose‐volumes for all six treatment plans (Pre_intensity modulated radiotherapy (IMRT), Pre_volumetric modulated arc theraphy (VMAT), composite rectum wall (CRW)_IMRT, CRW_VMAT, rectal wall (RW)_IMRT and RW_VMAT) compared to Mariados et al. pivotal trial pre‐ and post‐ SpaceOAR© hydrogel (SOH) results. 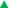, Mariados et al. Pre; 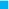, VMAT Pre; 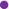, IMRT Pre; 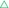, Mariados et al. Post; 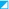, VMAT_CRW Post; 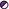, IMRT_CRW Post; 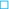, VMAT_RW Post; 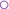, IMRT_RW Post.

Figure 2 depicts the mean rectal dose‐volume results for all six treatment plans generated in this retrospective study in relation to the pre‐ and post‐SOH results obtained in the pivotal SOH trial by Mariados et al. Even though different endpoints, optimization structures, and techniques were used to generate pre‐ and post‐SOH treatment plans in both of these studies, similar gains in rectal sparing were achieved. Gain in rectal sparing of 25% was considered clinically relevant as this gain was seen in RV70 Gy between use of three dimensional conformal radiotherapy (3D‐CRT) and IMRT for prostate cancer treatment with RV70 Gy being linked to high rectal toxicity.^24^ Mariados et al. and Song et al.^8^, ^10^ observed a 25% reduction in RV70 Gy occurring in 95.7% and 97.3% of patients respectively. The corresponding isodose equivalent in the hypofractionated regime is RV55 Gy and as such clinically relevant reduction of 25% was seen in 92% (12/13) of patients for RW plans and 85% (11/13) of patients for CRW plans. Furthermore, a 50% reduction of RV55 Gy was seen in 85% (11/13) of patients for all post‐SOH treatment plans.

The rectal dose in all six plans was compared to determine which optimization structure or treatment technique was most effective. The RW optimization structure was shown to result in significantly lower rectal doses than the CRW optimization structure (Table 5, Fig. 2) regardless of planning technique. Although the RW optimization structure was shown to be significantly better for rectal dose, the CRW structure achieved rectal doses similar to those published in other SOH studies such as Mariados et al.^8^ (Fig. 2). This trend is followed for all plans with the exception of RV60 Gy in both VMAT (*P* = 0.859) and IMRT (*P* = 0.890) plans and RV37 Gy in IMRT (*P* = 0.019) plans only. The latter has a p‐value which was close to the limit of statistically significant. Meanwhile, the former may be due to the near zero value of RV60 Gy metrics in post‐SOH treatment plans. In some patients, pre‐SOH plans experienced an RV60Gy of zero (IMRT: 6/13, VMAT: 4/13) and as such, there was limited room for improvement of this metric. It is important to note that the CRW structure offered slightly lower RV60 Gy in several patients (IMRT: 3/13, VMAT: 5/13) which caused RV60 Gy to be inconsistent with the trend seen in other dose metrics. The sparing offered by each respective post‐SOH treatment plan was comparable with the reduction seen in many SOH studies.^8^, ^10^, ^14^, ^15^, ^16^ The dose metrics used in the present study were all, except for one, relative to the total volume contoured. For consistency purposes, all target and OAR contours on the anonymized CT datasets were defined and peer‐reviewed by a small group of genitourinary radiation oncologists. The contoured volumes, summarized in Table 3, indicate no significant difference between pre‐ and post‐SOH implant except for the PTV. Since the PTV was an expansion of the CTV, any differences between pre‐ and post‐SOH CTV were augmented by the application of margins. On average, post‐SOH CTV volumes were 35 ± 8 cc while pre‐SOH CTV volumes were 34 ± 7 cc (*P* = 0.04). The post‐SOH CTV volumes were found to be 4.7% larger compared to pre‐SOH volumes after an average per patient ratio. The small, but almost statistically significant, difference in the CTV median volume was likely due to a combination of prostate edema from fiducial marker insertion, CTV contouring variation, and differences in the prostate appearance on the pre‐ and post‐SOH CT scans. The prostate edema effect has been well‐documented after brachytherapy seed implant.^25^ Intra‐ and inter‐observer variability of around 10%–18%^26^ have been documented for prostate contouring on CT. The pre‐SOH CTV structures were based on CT simulation while post‐SOH CTV incorporated both CT and MRI offering higher contrast for specific structures.

**Figure 3:**
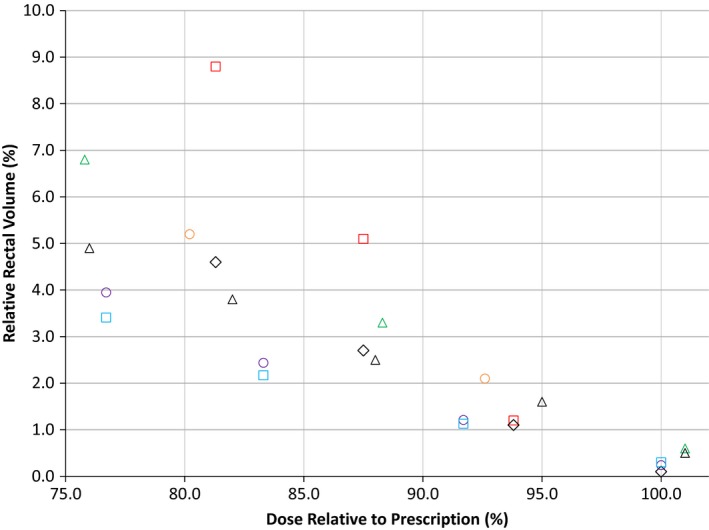
Mean volumetric modulated arc therapy and intensity modulated radiotherapy post‐SpaceOAR© hydrogel (SOH) rectal dose‐volumes achieved in this study with rectal wall compared to other published SOH studies, including Mariados et al. and Rajecki et al. 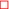, Song et al. Post; 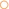, te Velde et al. Post; 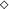, van Gysen et al. Post; 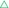, Mariados et al. Post; 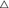, Rajecki et al. Post; 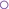, IMRT_RW Post; 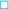, VMAT_RW Post.

Most SOH dosimetric studies contoured the rectum from the anus to the rectosigmoid junction^8^, ^10^, ^14^, ^15^, ^16^ with the exception of te Velde et al. which contoured the rectum wall 15 mm superiorly and inferiorly of the caudal or cranial CTV slice for optimization and plan evaluation purposes. As such, the rectum used to generate RW in this retrospective study was also contoured from anus to rectosigmoid junction in order to better compare our study to the current published literature. Mean post‐SOH rectal dose‐volumes from several studies are presented in Fig. 3 with the dose relative to the prescription. The RW VMAT and IMRT plans offered the lowest recorded mean rectal dose volumes in this study and were plotted in comparison. The observable differences in post‐SOH rectal dose volumes between published studies can be explained in part due to differences in absolute rectal and PTV volumes. Song et al.^10^ had the highest rectal dose volume of dosimetric studies published with dose volumes twice as high for the 80%–90% dose range when compared to van Gysen et al.^14^ However, patients in Song et al. had a 36% larger average rectum volume and an 8% larger average PTV volume compared to van Gysen et al. The larger rectum and PTV size likely resulted in more overlap between of the PTV and the rectum leading to higher rectum dose volumes. Mean rectal dose volumes reported for the RW optimization structure in this study are slightly lower than those reported in other dosimetric studies and more than 50% lower than van Gysen et al. for the same 80%–90% dose range. However, PTV volumes in this current study were 30% lower on average while rectum volumes were 15% higher, likely leading to lower PTV overlap with the rectum and therefore lower rectal dose volumes.

Bladder dose was evaluated using V60 Gy, V55 Gy, V50 Gy, V46 Gy, and V37 Gy metrics. Each treatment plan passed the clinical objectives, BWV46 Gy < 20% and BWV37 Gy < 40%. There were no statistically significant changes in bladder dose pre‐ to post‐SOH in VMAT or IMRT treatment plans consistent (*P* > 0.1) with other studies.^8^, ^10^, ^14^ However, VMAT treatment plans resulted in lower bladder dose compared to IMRT treatment plans (*P* < 0.007). Similarly, mean penile bulb dose was found to have no statistically significant difference between pre‐ and post‐SOH plans (*P* > 0.24) while VMAT treatment techniques yielded lower PB dose with an improvement ranging from 0.5%–1.0% (*P* < 0.04). Other studies reported higher pre‐ and post‐SOH mean PB dose while reporting a decrease in mean PB dose from pre‐ to post‐8 which is inconsistent with the results of this study.

Finally, the gradient measure (GM) and the conformity index (CI) indices were useful indicators of plan quality in addition to the OAR dose (Table 6). In Eclipse, GM was defined as the difference between the equivalent sphere radii of the prescription and 50% isodose lines while CI was defined as the volume enclosed by the prescription isodose surface divided by the target volume. VMAT plans had a statistically significant (*P* < 0.002) decrease in GM compared to IMRT while no change in CI was seen between plans. Although VMAT post‐SOH treatment plans offered lower bladder dose, mean penile bulb dose and lower GM, IMRT treatment plans were created two times more quickly and as such there must be consideration made to the planning time required.

**Table 6:** Gradient and conformity indices from six treatment plans

Plan/metric	Pre_IMRT	CRW_IMRT	RW_IMRT	Pre_VMAT	CRW_VMAT	RW_VMAT
Gradient measure (cm)	2.49 ± 0.37 (2.04–3.57)	2.39 ± 0.26 (2.11–2.89)	2.44 ± 0.23 (2.18–2.99)	1.65 ± 0.07 (1.52–1.76)	1.72 ± 0.08 (1.63–1.90)	1.66 ± 0.09 (1.52–1.84)
Conformity index	0.92 ± 0.05 (0.79–0.97)	0.91 ± 0.03 (0.86–0.97)	0.92 ± 0.03 (0.85–0.95)	0.90 ± 0.01 (0.88–0.91)	0.90 ± 0.01 (0.86–0.92)	0.92 ± 0.03 (0.86–0.99)

IMRT: intensity modulated radiotherapy; RW: rectal wall; VMAT: volumetric modulated arc therapy.

## 5 CONCLUSIONS

Rectal dose sparing greater than 25% was achieved in most post‐SpaceOAR© Hydrogel treatment plans generated in this planning study. The use of SpaceOAR© Hydrogel significantly reduced rectal dose regardless of optimization structure or treatment technique employed. The rectal wall optimization structure offered a statistically significant reduction in rectal dose compared to the CRW. There was no difference in rectal dose when using VMAT and IMRT treatment techniques, but VMAT offered lower bladder dose, mean penile bulb dose and gradient measure.

## CONFLICT OF INTEREST

No conflict of interest.
